# PDBe aggregated API: programmatic access to an integrative knowledge graph of molecular structure data

**DOI:** 10.1093/bioinformatics/btab424

**Published:** 2021-06-03

**Authors:** Sreenath Nair, Mihály Váradi, Nurul Nadzirin, Lukáš Pravda, Stephen Anyango, Saqib Mir, John Berrisford, David Armstrong, Aleksandras Gutmanas, Sameer Velankar

**Affiliations:** European Molecular Biology Laboratory, European Bioinformatics Institute (EMBL-EBI), Protein Data Bank in Europe, Wellcome Genome Campus, Hinxton CB10 1SA, UK; European Molecular Biology Laboratory, European Bioinformatics Institute (EMBL-EBI), Protein Data Bank in Europe, Wellcome Genome Campus, Hinxton CB10 1SA, UK; European Molecular Biology Laboratory, European Bioinformatics Institute (EMBL-EBI), Protein Data Bank in Europe, Wellcome Genome Campus, Hinxton CB10 1SA, UK; European Molecular Biology Laboratory, European Bioinformatics Institute (EMBL-EBI), Protein Data Bank in Europe, Wellcome Genome Campus, Hinxton CB10 1SA, UK; European Molecular Biology Laboratory, European Bioinformatics Institute (EMBL-EBI), Protein Data Bank in Europe, Wellcome Genome Campus, Hinxton CB10 1SA, UK; European Molecular Biology Laboratory, European Bioinformatics Institute (EMBL-EBI), Protein Data Bank in Europe, Wellcome Genome Campus, Hinxton CB10 1SA, UK; European Molecular Biology Laboratory, European Bioinformatics Institute (EMBL-EBI), Protein Data Bank in Europe, Wellcome Genome Campus, Hinxton CB10 1SA, UK; European Molecular Biology Laboratory, European Bioinformatics Institute (EMBL-EBI), Protein Data Bank in Europe, Wellcome Genome Campus, Hinxton CB10 1SA, UK; European Molecular Biology Laboratory, European Bioinformatics Institute (EMBL-EBI), Protein Data Bank in Europe, Wellcome Genome Campus, Hinxton CB10 1SA, UK; European Molecular Biology Laboratory, European Bioinformatics Institute (EMBL-EBI), Protein Data Bank in Europe, Wellcome Genome Campus, Hinxton CB10 1SA, UK

## Abstract

**Summary:**

The PDBe aggregated API is an open-access and open-source RESTful API that provides programmatic access to a wealth of macromolecular structural data and their functional and biophysical annotations through 80+ API endpoints. The API is powered by the PDBe graph database (https://pdbe.org/graph-schema), an open-access integrative knowledge graph that can be used as a discovery tool to answer complex biological questions.

**Availability and implementation:**

The PDBe aggregated API provides up-to-date access to the PDBe graph database, which has weekly releases with the latest data from the Protein Data Bank, integrated with updated annotations from UniProt, Pfam, CATH, SCOP and the PDBe-KB partner resources. The complete list of all the available API endpoints and their descriptions are available at https://pdbe.org/graph-api. The source code of the Python 3.6+ API application is publicly available at https://gitlab.ebi.ac.uk/pdbe-kb/services/pdbe-graph-api.

**Supplementary information:**

Supplementary data are available at *Bioinformatics* online.

## 1 Introduction

Macromolecular structural data are invaluable to derive a mechanistic understanding of molecular systems, and for potentially modulating them (Batool *et al.*, 2019; Metz *et al.*, 2012; Waman *et al.*, 2020). However, to take advantage of these data, it is important to place them in their biological context, where the atomic coordinates of molecular structures form only one part of the puzzle (Gerstein, 2000). The worldwide Protein Data Bank consortium (wwPDB), of which the Protein Data Bank in Europe (PDBe) is a founding member, manages the single worldwide archive of macromolecular structure data, the Protein Data Bank (Armstrong *et al.*, 2020; Berman *et al.*, 2003; wwPDB, 2019). In 2018, the PDBe team established a new resource, the PDBe-Knowledge Base (PDBe-KB, https://pdbe-kb.org) which integrates macromolecular structure data with their functional and biophysical annotations provided by members of the PDBe-KB consortium (Supplementary Table S1) (PDBe-KB, 2020).

The PDBe graph database, developed and maintained by the PDBe team, integrates core PDB data with value-added information from the weekly release cycle of PDBe (Armstrong *et al.*, 2020), UniProt mappings provided by SIFTS (Dana *et al.*, 2019) and functional annotations provided by the PDBe-KB consortium (PDBe-KB, 2020). This highly interconnected and rich knowledge graph allows the scientific community to make sophisticated queries to get datasets which would otherwise be labour-intensive to acquire.

We describe the implementation of the PDBe aggregated application programmatic interface (API) whose purpose is to facilitate programmatic data access through a comprehensive set of 80+ API endpoints that retrieve data from the PDBe graph database. We also demonstrate how the PDBe aggregated API can expedite the collation of a dataset which would otherwise require retrieving data from multiple data resources and performing extensive processing and aggregation.

## 2 Materials and methods

**Fig. 1.**
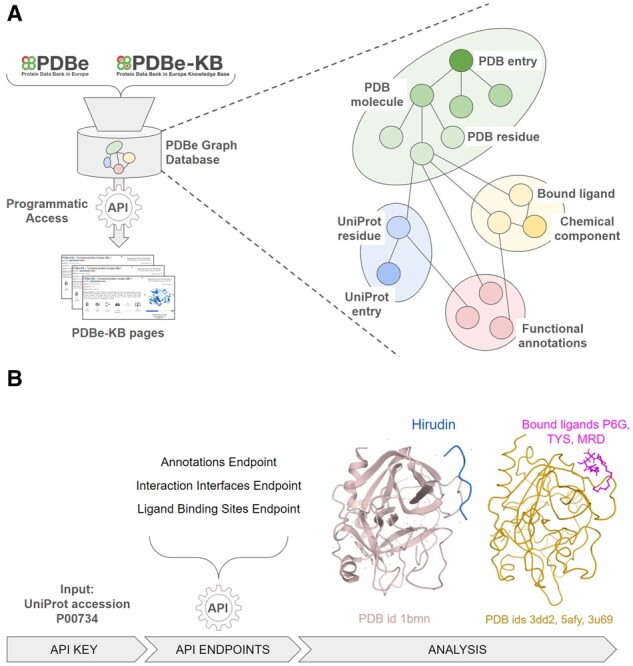
(**A**) An overview of how the PDBe aggregated API provides programmatic access to the PDBe knowledge graph, which combines data from PDBe and PDBe-KB. The graph data model of the underlying database provides a more intuitive representation of molecular entities and their relations. (**B**) A high-level schematic for a data retrieval procedure, starting from the UniProt accession of Thrombin (P00734). Users can combine data from multiple API endpoints, such as the annotations, interaction interfaces and ligand binding site endpoints to compile data on which ligand molecules bind to Thrombin at the same region as its natural inhibitor, Hirudin. A link to a step-by-step procedure is provided in the main text

A high-level overview of the PDBe graph infrastructure is displayed in Figure 1A. The graph database consolidates the core PDB data, residue-level mappings between PDB and UniProt using SIFTS data and functional and biophysical annotations provided by the PDBe-KB partner resources (Supplementary Table S1). The data are represented as a graph, i.e. nodes and edges, with several nodes describing macromolecular structures (e.g. PDB entry nodes are connected to PDB chain nodes, which are in turn connected to PDB residue nodes), while other nodes represent data related to protein sequences (i.e. UniProt accessions), small molecules and functional annotations. It is important to note that nodes such as PDB residues are directly connected to their respective UniProt residues, as well as any small molecules they interact with, and any functional annotations they might have. This data model and graph encoding facilitates data mining, for example retrieving a complete list of small molecules interacting with a particular UniProt residue in any mapped PDB structure, while also filtering for only those UniProt residues which form catalytic sites. The complete data schema is documented at https://pdbe.org/graph-schema.

Users can programmatically access data by sending GET requests to a rich set of API endpoints. These endpoints are used by both PDBe and PDBe-KB pages, necessitating that the API is maintained, up-to-date and reliably accessible. For example, the endpoint https://www.ebi.ac.uk/pdbe/graph-api/mappings/best_structures/P00734 (where ‘P00734’ is a UniProt accession) returns a list of PDB entries mapped to the given UniProt accession. The returned list of PDBs is sorted by the combination of coverage of the protein (i.e. the segment of the sequence covered by a PDB structure), the resolution of the structure and the overall data quality, with the ‘best’ representative PDB structure first in the list. Other endpoints serve information about small molecules and their interactions with a given macromolecule, such as https://www.ebi.ac.uk/pdbe/graph-api/pdbe_pages/binding_sites/4ud9/1 (where ‘4ud9’ is the PDB identifier and ‘1’ is the entity identifier for the macromolecule) which returns detailed information on the ligands observed to be interacting with the particular macromolecule in a PDB structure. The complete set of 80+ endpoints, their data schema and examples are documented at https://pdbe.org/graph-api.

## 3 Discussion and conclusion

The strength of the PDBe aggregated API stems from the underlying highly interconnected graph representation of the data as nodes and the logical connections between pieces of information which are encoded as edges. This allows the users to perform complex queries that would otherwise be computationally expensive to perform using more traditional relational data models.

We demonstrate one such example in Figure 1B. Starting from the UniProt accession of Thrombin, users can programmatically combine the output of two API endpoints to identify all the amino acid residues of a protein that are putative ligand binding sites, according to PDBe-KB partner resources such as P2rank (Krivák and Hoksza, 2018) and are also potentially druggable pockets according to canSAR (Coker *et al.*, 2019), while additionally being observed on macromolecular interaction interfaces, according to the PISA software (Krissinel and Henrick, 2007). Thrombin (UniProt accession P00734) has several residues that satisfy these criteria when programmatically parsing and combining data from the annotations API endpoint (https://www.ebi.ac.uk/pdbe/graph-api/uniprot/annotations/P00734) and the interaction interfaces endpoint (https://www.ebi.ac.uk/pdbe/graph-api/uniprot/interface_residues/P00734). For example, the anti-coagulant peptide, Hirudin (variant-1) interacts with the Thrombin region between residues GLU388 and GLY591, and this region also contains 9 putative ligand binding sites and druggable pockets according to annotations from P2rank and canSAR. Information such as this can help researchers design small molecules that may disrupt the function of Thrombin. The API endpoint https://www.ebi.ac.uk/pdbe/graph-api/uniprot/ligand_sites/P00734 lists all the binding sites for ligands observed in PDB structures of Thrombin, and there are 281 small molecules that interact with the same 9 residues, which are also involved in the interactions of Thrombin with Hirudin. We display the Thrombin-Hirudin complex and three of these ligands, Hexaethylene glycol (P6G), Sulfotyrosine (TYS) and (4R)-2-Methylpentane-2,4-diol (MRD) on the right-hand side of Figure1B.

A step-by-step guide for reproducing this data retrieval procedure using Python3 is available as a Jupyter Notebook via CodeOcean at https://doi.org/10.24433/CO.4804830.v1.

The interconnected nature of the graph representation of structural data and functional annotations allows further complex queries, such as: Can we find small molecules which have the same scaffold and interact with the same binding sites? Are these molecules interacting with residues annotated as catalytic sites? Are any of these ligands annotated as drug-like molecules? In addition to ligand-related queries, the API can also help with macromolecular complex-related queries, such as: Can we find macromolecular interaction interfaces which have highly conserved residues and which are also annotated to be structurally flexible? Performing such queries would often require reliance on the collation of data from multiple resources, whereas the PDBe aggregated API and the underlying graph database makes such queries accessible to the broader scientific community.

Powered by an up-to-date repository of all the structural data of the Protein Data Bank, and with an ever-growing set of functional and biophysical annotations, the PDBe aggregated API is a powerful research tool that can provide quick, easy and reliable access to data for answering complex scientific questions.

## Funding

This work was supported by Biotechnology and Biological Sciences Research Council via the 3D-Gateway [BB/T01959X/1] and FunPDBe [BB/P024351/1] grants, the Wellcome Trust [104948] and the European Molecular Biology Laboratory-European Bioinformatics Institute.

*Conflict of Interest*: none declared.

## Supplementary Material

btab424_Supplementary_Data
