# Supplementary material for: PDBe aggregated API: programmatic access to an integrative knowledge graph of molecular structure data
Source: Bioinformatics. 2021 Jun 3;37(21):3950–2. doi: 10.1093/bioinformatics/btab424 (PMC8570819; doi:10.1093/bioinformatics/btab424)
Supplement: btab424_Supplementary_Data [file btab424_supplementary_data.docx]

| **Data provider** | **Annotation Type** | **Reference** |
| --- | --- | --- |
| 14-3-3-pred | Predicted 14-3-3-binding sites | ([Madeira et al., 2015](#_ENREF_10)) |
| 3D-Complex | Interaction interface annotations | ([Levy, Pereira-Leal, Chothia, & Teichmann, 2006](#_ENREF_9)) |
| 3D-LigandSite | Putative ligand binding sites | ([Wass, Kelley, & Sternberg, 2010](#_ENREF_20)) |
| AKID | Predicted kinase-specific phosphorylation | ([Parca et al., 2019](#_ENREF_12)) |
| CaMKinet | Calmodulin-dependent kinase annotations | (*in preparation*) |
| canSAR | Predicted druggable pockets | ([Coker et al., 2019](#_ENREF_3)) |
| CATH-FunSites | Predicted functional sites | ([Ashford, Pang, Moya-Garcia, Adeyelu, & Orengo, 2019](#_ENREF_1)) |
| ChannelsDB | Predicted molecular channels | ([Pravda et al., 2018](#_ENREF_13)) |
| Covalentizer | Potential covalent binder sites | (*in preparation*) |
| DEPTH | Residue depth | ([Tan, Nguyen, Patel, Varadarajan, & Madhusudhan, 2013](#_ENREF_18)) |
| DynaMine | Predicted backbone flexibility | ([Cilia, Pancsa, Tompa, Lenaerts, & Vranken, 2013](#_ENREF_2)) |
| EFoldMine | Predicted early folding residues | ([Raimondi, Orlando, Pancsa, Khan, & Vranken, 2017](#_ENREF_15)) |
| FireProt DB | Effect on the stability of point mutations | ([Stourac et al., 2021](#_ENREF_17)) |
| FoldX | Effect on the stability of point mutations | ([Delgado, Radusky, Cianferoni, & Serrano, 2019](#_ENREF_5)) |
| KinCore | Conformational annotations of kinases | (*in preparation*) |
| KnotProt | Topology annotations | ([Dabrowski-Tumanski et al., 2019](#_ENREF_4)) |
| M-CSA | Catalytic sites | ([Ribeiro et al., 2018](#_ENREF_16)) |
| MetalPDB | The biological relevance of metal ions | ([Putignano, Rosato, Banci, & Andreini, 2018](#_ENREF_14)) |
| Missense3D | Effect on the stability of point mutations | ([Ittisoponpisan et al., 2019](#_ENREF_6)) |
| P2rank | Predicted ligand binding sites | ([Krivak & Hoksza, 2018](#_ENREF_8)) |
| POPScomp | Accessible surface area | ([Kleinjung & Fraternali, 2005](#_ENREF_7)) |
| ProKinO | Protein kinase annotations | ([McSkimming et al., 2015](#_ENREF_11)) |
| WEBnm@ | Flexibility predictions | ([Tiwari et al., 2014](#_ENREF_19)) |

**Supplementary Table 1 - PDBe-KB partner resources providing annotations**

**References**

Ashford, P., Pang, C. S. M., Moya-Garcia, A. A., Adeyelu, T., & Orengo, C. A. (2019). A CATH domain functional family based approach to identify putative cancer driver genes and driver mutations. *Sci Rep, 9*(1), 263. doi: 10.1038/s41598-018-36401-4

Cilia, E., Pancsa, R., Tompa, P., Lenaerts, T., & Vranken, W. F. (2013). From protein sequence to dynamics and disorder with DynaMine. *Nat Commun, 4*, 2741. doi: 10.1038/ncomms3741

Coker, E. A., Mitsopoulos, C., Tym, J. E., Komianou, A., Kannas, C., Di Micco, P., . . . Al-Lazikani, B. (2019). canSAR: update to the cancer translational research and drug discovery knowledgebase. *Nucleic Acids Res, 47*(D1), D917-D922. doi: 10.1093/nar/gky1129

Dabrowski-Tumanski, P., Rubach, P., Goundaroulis, D., Dorier, J., Sulkowski, P., Millett, K. C., . . . Sulkowska, J. I. (2019). KnotProt 2.0: a database of proteins with knots and other entangled structures. *Nucleic Acids Res, 47*(D1), D367-D375. doi: 10.1093/nar/gky1140

Delgado, J., Radusky, L. G., Cianferoni, D., & Serrano, L. (2019). FoldX 5.0: working with RNA, small molecules and a new graphical interface. *Bioinformatics, 35*(20), 4168-4169. doi: 10.1093/bioinformatics/btz184

Ittisoponpisan, S., Islam, S. A., Khanna, T., Alhuzimi, E., David, A., & Sternberg, M. J. E. (2019). Can Predicted Protein 3D Structures Provide Reliable Insights into whether Missense Variants Are Disease Associated? *J Mol Biol, 431*(11), 2197-2212. doi: 10.1016/j.jmb.2019.04.009

Kleinjung, J., & Fraternali, F. (2005). POPSCOMP: an automated interaction analysis of biomolecular complexes. *Nucleic Acids Res, 33*(Web Server issue), W342-346. doi: 10.1093/nar/gki369

Krivak, R., & Hoksza, D. (2018). P2Rank: machine learning based tool for rapid and accurate prediction of ligand binding sites from protein structure. *J Cheminform, 10*(1), 39. doi: 10.1186/s13321-018-0285-8

Levy, E. D., Pereira-Leal, J. B., Chothia, C., & Teichmann, S. A. (2006). 3D complex: a structural classification of protein complexes. *PLoS Comput Biol, 2*(11), e155. doi: 10.1371/journal.pcbi.0020155

Madeira, F., Tinti, M., Murugesan, G., Berrett, E., Stafford, M., Toth, R., . . . Barton, G. J. (2015). 14-3-3-Pred: improved methods to predict 14-3-3-binding phosphopeptides. *Bioinformatics, 31*(14), 2276-2283. doi: 10.1093/bioinformatics/btv133

McSkimming, D. I., Dastgheib, S., Talevich, E., Narayanan, A., Katiyar, S., Taylor, S. S., . . . Kannan, N. (2015). ProKinO: a unified resource for mining the cancer kinome. *Hum Mutat, 36*(2), 175-186. doi: 10.1002/humu.22726

Parca, L., Ariano, B., Cabibbo, A., Paoletti, M., Tamburrini, A., Palmeri, A., . . . Helmer-Citterich, M. (2019). Kinome-wide identification of phosphorylation networks in eukaryotic proteomes. *Bioinformatics, 35*(3), 372-379. doi: 10.1093/bioinformatics/bty545

Pravda, L., Sehnal, D., Svobodova Varekova, R., Navratilova, V., Tousek, D., Berka, K., . . . Koca, J. (2018). ChannelsDB: database of biomacromolecular tunnels and pores. *Nucleic Acids Res, 46*(D1), D399-D405. doi: 10.1093/nar/gkx868

Putignano, V., Rosato, A., Banci, L., & Andreini, C. (2018). MetalPDB in 2018: a database of metal sites in biological macromolecular structures. *Nucleic Acids Res, 46*(D1), D459-D464. doi: 10.1093/nar/gkx989

Raimondi, D., Orlando, G., Pancsa, R., Khan, T., & Vranken, W. F. (2017). Exploring the Sequence-based Prediction of Folding Initiation Sites in Proteins. *Sci Rep, 7*(1), 8826. doi: 10.1038/s41598-017-08366-3

Ribeiro, A. J. M., Holliday, G. L., Furnham, N., Tyzack, J. D., Ferris, K., & Thornton, J. M. (2018). Mechanism and Catalytic Site Atlas (M-CSA): a database of enzyme reaction mechanisms and active sites. *Nucleic Acids Res, 46*(D1), D618-D623. doi: 10.1093/nar/gkx1012

Stourac, J., Dubrava, J., Musil, M., Horackova, J., Damborsky, J., Mazurenko, S., & Bednar, D. (2021). FireProtDB: database of manually curated protein stability data. *Nucleic Acids Res, 49*(D1), D319-D324. doi: 10.1093/nar/gkaa981

Tan, K. P., Nguyen, T. B., Patel, S., Varadarajan, R., & Madhusudhan, M. S. (2013). Depth: a web server to compute depth, cavity sizes, detect potential small-molecule ligand-binding cavities and predict the pKa of ionizable residues in proteins. *Nucleic Acids Res, 41*(Web Server issue), W314-321. doi: 10.1093/nar/gkt503

Tiwari, S. P., Fuglebakk, E., Hollup, S. M., Skjaerven, L., Cragnolini, T., Grindhaug, S. H., . . . Reuter, N. (2014). WEBnm@ v2.0: Web server and services for comparing protein flexibility. *BMC Bioinformatics, 15*, 427. doi: 10.1186/s12859-014-0427-6

Wass, M. N., Kelley, L. A., & Sternberg, M. J. (2010). 3DLigandSite: predicting ligand-binding sites using similar structures. *Nucleic Acids Res, 38*(Web Server issue), W469-473. doi: 10.1093/nar/gkq406
